# Predictors for early‐onset psychotic symptoms in patients newly diagnosed with Parkinson's disease without psychosis at baseline: A 5‐year cohort study

**DOI:** 10.1111/cns.14651

**Published:** 2024-03-03

**Authors:** Jing Chen, Baoyu Chen, Danhua Zhao, Xiaotong Feng, Qi Wang, Yuan Li, Junyi Chen, Chaobo Bai, Xintong Guo, Xiaoyu He, Lin Zhang, Junliang Yuan

**Affiliations:** ^1^ Department of Neurology Peking University Sixth Hospital, Peking University Institute of Mental Health, NHC Key Laboratory of Mental Health (Peking University), National Clinical Research Center for Mental Disorders (Peking University Sixth Hospital), Peking University Beijing China; ^2^ Department of Neurology, PF Center of Excellence, UC Davis Medical Center, UC Davis School of Medicine Sacramento California USA

**Keywords:** Aβ42: Total‐tau, gastrointestinal autonomic dysfunction, Parkinson's disease, psychosis, RBD

## Abstract

**Aims:**

To investigate the risk factors for early‐onset psychosis in Parkinson's disease (PD) in a cohort of patients from the Parkinson's Progression Markers Initiative.

**Methods:**

Longitudinal data on motor and non‐motor features, dopamine transporter (DAT) imaging, and cerebrospinal fluid (CSF) measurements were collected. The survival probability of psychotic symptoms, potential risk factors for psychosis development over a 5‐year follow‐up period, and the performance of the prediction model were evaluated.

**Results:**

Among the 338 newly diagnosed patients with PD, 83 developed psychotic symptoms. Gastrointestinal autonomic dysfunction, presence of probable rapid‐eye‐movement sleep behavior disorder, and the ratio Aβ42: total‐tau could independently predict onset of psychosis in PD (hazard ratio (*HR*) = 1.157, 95% confidence interval (*CI*) 1.022–1.309, *p* = 0.021, *HR* = 2.596, 95% *CI* 1.287–5.237, *p* = 0.008, and *HR* = 0.842, 95% *CI* 0.723–0.980, *p* = 0.027, respectively). The combined model integrating baseline clinical predictors, DAT imaging, and CSF measurements achieved better sensitivity than the clinical predictors alone (area under the curve = 0.770 [95% *CI* 0.672–0.868] vs. 0.714 [95% *CI* 0.625–0.802], *p* = 0.098).

**Conclusion:**

We identified clinical and CSF predictors of early‐onset psychosis in patients with PD. Our study provides evidence and implications for prognostic stratification and therapeutic approaches for PD psychosis.

## Graphical abstract

Greater gastrointestinal autonomic dysfunction, presence of pRBD, and Aβ42: total‐tau ratio could independently predict early onset of psychotic symptoms in newly diagnosed PD (diagnosed with PD for 2 years or less at screening visit) with no psychosis at baseline.

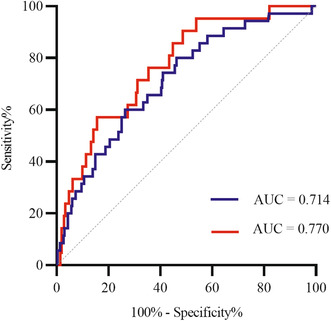

## 1 INTRODUCTION

Parkinson's disease (PD) affects approximately 6.1 million people worldwide.^1^ PD psychosis, a spectrum of illusions/hallucinations and delusions, is common at different disease stages, with a cumulative prevalence of 60%.^2^, ^3^ Psychosis symptoms are associated with an increased risk of dementia, increased caregiver burden, and higher mortality.^4^, ^5^, ^6^ The Parkinson's Progression Markers Initiative (PPMI) study reported that the prevalence of psychosis increased from 3% at PD diagnosis to 10% at 2‐year follow‐up.^7^ Earlier risk factor stratification for PD psychosis could be helpful for prognosticating the disease course and implementing appropriate interventions in the early stages of PD.

Many previous studies have identified various risk factors for developing psychosis in PD, including excessive daytime sleepiness (EDS), older age at onset of PD, longer disease duration, depression, use of anti‐parkinsonian medication, female sex, dyskinesia, rapid‐eye‐movement sleep behavior disorder (RBD), cognitive impairment, and autonomic dysfunction; however, most of these results were based on patients in the advanced stages of PD.^3^, ^4^, ^8^, ^9^, ^10^, ^11^, ^12^, ^13^, ^14^, ^15^ Longitudinal studies on early‐onset psychosis in patients newly diagnosed with PD are still lacking. Three studies included in the PPMI focused on the risk factors for early‐onset psychosis in PD, and the findings of autonomic dysfunction and the presence of EDS and RBD were risk factors for early‐onset psychosis in PD. However, these results were from a limited number of patients or restricted follow‐up periods owing to data collection time constraints or prior study designs.^10^, ^11^, ^16^ To date, there have been still no exact studies to assess the effect of specific autonomic domains on the development of early‐onset PD psychosis.

This study first aimed to explore the predictors of early‐onset psychosis in PD by testing comprehensive data on clinical variables, dopamine transporter (DAT)‐imaging, and cerebrospinal fluid (CSF) measurements. Second, we aimed to determine whether specific autonomic domains are associated with the development of early‐onset psychosis in PD.

## 2 METHODS

### 2.1 Study design and participants

The PPMI is an ongoing observational, international, multicenter study aimed at identifying biomarkers of PD progression in participants with early untreated (de novo) PD (diagnosed within 2 years). Data used in this study were collected from the PPMI cohort database. Details of the eligibility criteria are published on the PPMI website (http://www.ppmi‐info.org). Each PPMI participant provided written informed consent, and the PPMI study was approved by the institutional board of each study site. Our study was conducted using PPMI data collected between January 2011 and November 2017, and all patients were followed up for 5 years.

The subjects of the study were patients newly diagnosed with PD (diagnosed with PD for 2 years or less at screening visit), who were followed up for 5 years and had no psychotic symptoms at baseline. Patients with PD meeting the following criteria were included: (1) had annual follow‐up assessments for 5 years; (2) had scores on item 1.2 in the Movement Disorder Society‐Sponsored Revision of the Unified Parkinson's Disease Rating Scale (MDS‐UPDRS) at baseline and at each annual follow‐up; and (3) a score of 0 for item 1.2 in the MDS‐UPDRS at baseline. Patients who underwent deep brain stimulation surgery during the observation period were excluded from the study.

### 2.2 Assessment of psychotic symptoms

Psychotic symptoms were assessed using item 1.2 of the MDS‐UPDRS. The MDS‐UPDRS Part I scale assesses the non‐motor impact of PD on daily living experiences using 13 questions. Among these questions, item 1.2 evaluates hallucinations and psychotic behavior over the past week and was rated as 0 = normal, 1 = slight, 2 = mild, 3 = moderate, or 4 = severe. Slight hallucinations (score of 1) involved illusions or non‐formed hallucinations that patients could recognize without loss of insight, whereas mild hallucinations (score of 2) involved formed hallucinations independent of environmental stimuli and no loss of insight. Patients with moderate hallucinations (score 3) experienced hallucinations with a loss of insight. Severe hallucinations (score of 4) are evaluated when patients with PD have delusions or paranoia.

Patients with any score >0 for item 1.2 in the MDS‐UPDRS were considered to have psychotic symptoms. The study defined four groups as follows: PD‐Psy0 included patients with a score of 0 at each annual assessment on item 1.2; PD‐Psy1 included patients with any score >0 on item 1.2 at only one assessment during the 5‐year follow‐up; PD‐Psy1+ included patients with any score >0 at one or more assessments on item 1.2; and PD‐Psy2+ included patients with any score >0 at two or more assessments on item 1.2. It is worth noting that the assessment window of 1 week for psychotic symptoms used in the PPMI study is inconsistent with the criteria for PD psychosis, which requires the presence of recurrent or continuous psychotic symptoms for at least 1 month. To improve specificity, this study defined a score greater than 0 at two or more assessments as the outcome when exploring the risk factors for psychotic symptoms in PD.

### 2.3 Candidate predictors

Demographic and clinical characteristics included in this study were age at baseline, sex, and duration of PD. Motor symptoms were assessed using the MDS‐UPDRS part III (MDS‐UPDRS III) scores, Hoehn and Yahr (H&Y) stage, and tremor‐dominant/postural instability and gait difficulty/indeterminate classification.^17^ The global cognitive function was assessed using the Montreal Cognitive Assessment (MoCA). Cognitive domains were assessed using a battery of neuropsychological tests, including the Hopkins Verbal Learning Test (HVLT) total recall and HVLT recognition discrimination index (HVLT RDI) for verbal memory, the Benton Judgment of Line Orientation (BJLO) for visuospatial function, Letter‐Number Sequencing (LNS) and the Semantic (animal) fluency Test for executive function/working memory, and the Symbol‐Digit Modalities Test for attention/processing speed. Depression was assessed using the 15‐item Geriatric Depression Scale. Excessive daytime sleepiness (EDS) was assessed using the Epworth Sleepiness Scale, with a score ≥10 indicating the presence of EDS.^18^ RBD was assessed using the RBD Screening Questionnaire (RBDSQ). According to the previous study, a score ≥1 on question 6 of the RBDSQ indicated the presence of probable RBD (pRBD).^19^, ^20^ Olfactory function was assessed using the University of Pennsylvania Smell Identification Test (UPSIT). Autonomic function was evaluated using the Scale for Outcomes in Parkinson's Disease for Autonomic Symptoms (SCOPA‐AUT). The total score and the scores for specific domains, including the gastrointestinal (GI), urinary, cardiovascular, thermoregulatory, sexual, and pupillomotor domains, were calculated. In addition, we included *APOE* ε4 status (ε4 homozygous, heterozygous, or negative) and *GBA* mutation status. We assessed peripheral inflammation using neutrophil count, lymphocyte count, and lymphocyte‐to‐neutrophil ratio in the peripheral blood.

### 2.4 DAT imaging

Details of the PPMI DAT imaging procedures and processing methods for calculating the calculation of striatal binding ratio (SBR) are available on the study website (http://www.ppmi‐info.org/). We included DAT imaging data for mean caudate and putaminal uptake relative to uptake in the occipital area and asymmetry of caudate and putaminal uptake (side with the highest divided by side with the lowest uptake).

### 2.5 CSF findings

The details of the PPMI CSF sampling and analysis procedures are described on the study website (http://www.ppmi‐info.org/). We evaluated CSF for Aβ_42_, total tau, phosphorylated tau181, α‐synuclein, and the calculated ratio of Aβ_42_ to total tau (Aβ_42_:t‐tau).

### 2.6 Statistical analysis

The Kolmogorov‐Smirnov test was used to assess the normality of the data. Normally distributed continuous variables are presented as mean ± standard deviation. Non‐normally distributed continuous variables were presented as medians and interquartile ranges. Categorical variables are expressed as percentages. For continuous variables, one‐way analysis of variance (ANOVA) (with Bonferroni post‐hoc test) was used to compare three groups if the group variances were homogeneous or Welch's analysis of variance was performed in the presence of heterogeneity, and the independent sample *t‐*test was used to compare two groups for normally distributed variables or the Mann‐Whitney *U* test for non‐normally distributed variables. For categorical variables, the chi‐square or Fisher's exact tests were performed to compare the groups. We first used univariate Cox regression proportional hazards models to identify possible risk factors for the development of psychotic symptoms in patients with PD during the 5‐year follow‐up period. Variables with values of *p* < 0.1 in the univariate COX regression analysis and no high correlation (*r* > 0.5) with each other were included in a multivariate COX regression proportional hazards model with a forward LR approach. The correlation of variables is tested using the Pearson correlation test. Kaplan‐Meier curves were used to display the survival probability of psychotic symptoms, with a log‐rank test to compare differences. Receiver operating characteristic curves were drawn, and areas under the curve (*AUC*) were calculated to estimate prediction accuracy. In addition, we explored the risk factors for developing psychotic symptoms at one or more assessments, in the same manner as described in the primary outcome analyses.

A two‐sided *p* < 0.05 was considered statistically significant. Statistical analysis was performed using IBM SPSS version 26 and GraphPad Prism version 9.

## 3 RESULTS

### 3.1 Psychosis outcome

**FIGURE 1:**
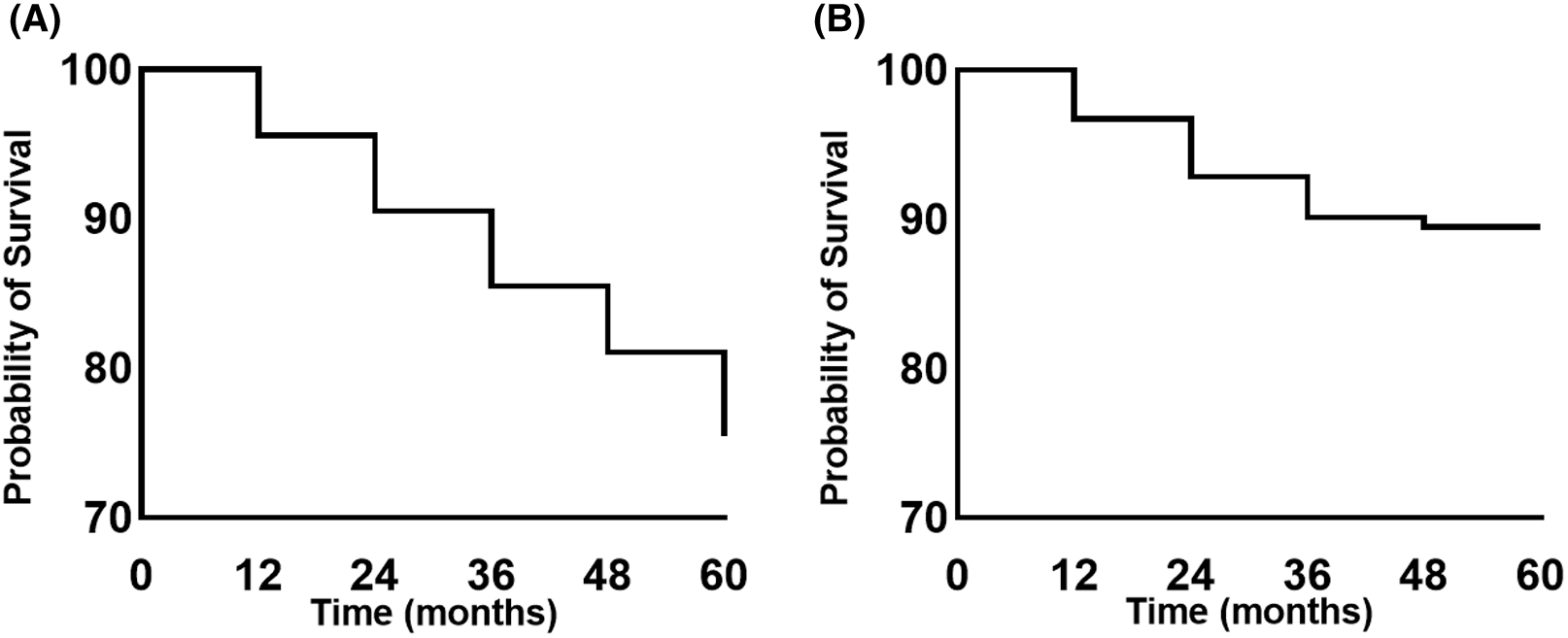
Kaplan‐Meier curves displaying risk of psychotic symptoms in newly diagnosed PD patients for 5 years. (A) Outcome refers to a score greater than 0 on item 1.2 of the MDS‐UPDRS at one or more assessments. (B) Outcome refers to a score greater than 0 on item 1.2 of the MDS‐UPDRS at two or more assessments. MDS‐UPDRS, Movement Disorders Society‐Unified Parkinson's Disease Rating Scale; PD, Parkinson's disease.

A total of 353 newly diagnosed patients with PD completed the 5‐year follow‐up and had answered item 1.2 of the MDS‐UPDRS assessment each year. Fifteen individuals with psychotic symptoms (any score >0 on item 1.2) at baseline were excluded from the study. Finally, 338 participants were included in the study. Among them, 83 developed psychotic symptoms during the 5‐year follow‐up period. The cumulative incidence of reporting psychotic symptoms at one or more assessment increased over time, with rates of 4.4%, 9.5%, 14.5%, 18.9%, and 24.6% at the 1‐, 2‐, 3‐, 4‐, and 5‐year follow‐up, respectively (Figure 1A). The cumulative incidence of reporting psychotic symptoms at two or more assessments was 2.4%, 5.3%, 8.9%, and 9.8% at the 2‐, 3‐, 4‐, and 5‐year follow‐ups, respectively (Figure 1B).

Among the patients who reported psychotic symptoms, approximately 11 of 83 (13.3%) consistently reported psychotic symptoms at each subsequent visit, whereas 18 of 83 (21.7%) first reported psychotic symptoms at their last follow‐up in the fifth year. It was common for patients to report psychotic symptoms at one follow‐up visit and none at subsequent visits (65.1%, 54 of 83). There were 146 positive events in 83 participants during the 5‐year follow‐up, including 123 events of an item 1.2 score of 1 in 76 patients, 15 events of an item 1.2 score of 2 in 14 patients, four events of an item 1.2 score of 3 in 4 patients, and four events of an item 1.2 score of 4 in 2 patients.

### 3.2 Clinical characteristics of PD patients with psychosis

As shown in Table 1, at baseline, patients with PD who developed psychotic symptoms during the 5‐year follow‐up period were more likely to have the H&Y‐2 type; higher scores on the MDS‐UPDRS III and SCOPA‐AUT; and lower scores on the UPSIT, HVLT RDI, and LNS (*p* < 0.05). However, no differences were found in GBA status, APOE status, or peripheral inflammation biomarkers (Tables S1–S4).

**TABLE 1:** Baseline demographic and clinical characteristics of patients with PD.

	PD‐Psy0 (*n* = 255)	PD‐Psy1 (*n* = 48)	PD‐Psy2+ (*n* = 35)	*p* value
Age, years	61.0 (9.9)	61.9 (8.8)	62.5 (8.2)	0.630
Male, *n* (%)	157 (61.6%)	25 (53.1%)	26 (74.3%)	0.121
Disease duration, years	4.0 (2.0–8.0)	2.0 (1.0–5.0)	4.0 (2.0–8.8)	0.293
MDS‐UPDRS III score	18.0 (14.0–24.0)	20.0 (15.5–24.0)	21.0 (16.0–25.0)	0.018* (Psy0 < Psy1, Psy0 < Psy2+)
H&Y (ON)				
H&Y‐1	123 (48.2%)	20 (43.5%)	10 (28.6%)	
H&Y‐2	130 (51.0%)	26 (56.5%)	24 (68.6%)	
H&Y‐3	2 (0.8%)	0 (0.0%)	1 (2.9%)	0.166
Motor subtype				
TD	197 (77.3%)	33 (68.8%)	23 (65.7%)	0.383
PIGD	48 (18.8%)	11 (22.9%)	10 (28.6%)	
Indeterminate	10 (3.9%)	4 (8.3%)	2 (5.7%)	
Cognitive function				
MoCA score	27.0 (26.0–29.0)	28.5 (26.3–29.8)	27.5 (26.0–29.0)	0.530
HVLT total recall	46.3 (10.5)	45.3 (11.2)	44.0 (10.8)	0.450
HVLT RDI	47.0 (38.0–53.5)	45.5 (37.0–52.0)	40.0 (36.0–37.0)	0.029* (Psy0 > Psy1, Psy0 > Psy2+)
LNS score	12.0 (10.0–13.0)	12.0 (10.3–13.0)	10.50 (9.3–11.0)	0.088 (Psy0 > Psy2+)
SDMT score	45.0 (39.0–51.0)	45.5 (37.2–50.0)	46.7 (43.8–47.9)	0.698
SFT score	21.0 (18.0–25.0)	21.0 (17.0–25.8)	20.0 (18.3–23.0)	0.909
BJLO score	28.0 (24.0–29.0)	27.0 (22.0–30.0)	26.0 (19.0–29.5)	0.195
ESS score	6.0 (4.0–8.0)	5.5 (3.0–10.0)	6.5 (4.0–6.0)	0.183
GDS‐15 score	5.0 (4.0–6.0)	5.0 (5.0–6.0)	5.0 (4.3–6.0)	0.269
pRBD, *n* (%)	52 (20.4%)	17 (20.7%)	13 (37.1%)	0.014* (Psy0 < Psy1, Psy0 < Psy2+)
SCOPA‐AUT				
Total score	11.6 (6.0–16.0)	12.6 (5.0–21.8)	14.7 (9.3–18.3)	0.045* (Psy0 < Psy2+)
Gastrointestinal	1.0 (0.0–3.0)	2.0 (0.0–4.0)	3.5 (2.0–5.0)	< 0.001** (Psy0 < Psy1, Psy0 < Psy2+)
Urinary	4.0 (2.0–5.0)	5.0 (2.0–6.8)	4.5 (3.0–7.0)	0.417
Cardiovascular	0.0 (0.0–1.0)	0.5 (0.0–1.0)	0.0 (0.0–1.0)	0.012* (Psy0 < Psy1)
Thermoregulatory	1.0 (0.0–2.0)	1.0 (0.0–2.0)	1.0 (0.0–2.0)	0.244
Sexual	1.0 (0.0–3.0)	1.0 (0.0–4.3)	2.5 (0.0–4.0)	0.911
Pupillomotor	0.0 (0.0–1.0)	0.0 (0.0–1.0)	0.0 (0.0–1.0)	0.042* (Psy0 < Psy2+)
UPSIT score	23.2 (8.1)	21.6 (8.3)	19.5 (9.3)	0.030* (Psy0 > Psy2+)

*Note*: Continuous variables were presented as mean ± standard deviation (SD) or median and interquartile range (IQR). Categorical variables were presented as numbers and percentages. For variables presented as mean (SD), one‐way ANOVA was used. For variables presented as number (percent), chi‐square test was used. For variable presented as median (IQR), Kruskal‐Wallis test was used.

Abbreviations: BJLO, Benton Judgment of Line Orientation; ESS, Epworth Sleepiness Scale; GDS, Geriatric Depression Scale; H&Y, Hoehn and Yahr stage; HVLT, Hopkins Verbal learning Test; LNS, Letter Number Sequencing; MDS‐UPDRS, Movement Disorders Society‐Unified Parkinson's Disease Rating Scale; MoCA, Montreal Cognitive Assessment; PD, Parkinson's disease; PD‐Psy0, PD patients with a score of 0 for item 1.2 in the MDS‐UPDRS for each annual assessment during the 5‐year follow‐up period; PD‐Psy1, PD patients with any score >0 at only one assessment; PD‐Psy2+, PD patients with any score >0 at two or more assessments; PIGD, postural instability/gait difficulty; pRBD, probable rapid‐eye‐movement sleep behavior disorder; RDI, Recognition Discrimination Index; SCOPA‐AUT, Scales for Outcomes in Parkinson's Disease‐Autonomic symptoms; SDMT, Symbol Digit Modalities Test; SFT, Semantic (animal) fluency Test; TD, tremor dominant; UPSIT, University of Pennsylvania Smell Inventory Test.

* *p* < 0.05.

** *p* < 0.01.

At the final assessment in the fifth year, patients with PD with psychotic symptoms were more likely to have pRBD and lower scores on the MoCA, HVLT RDI, and SCOPA‐AUT (total and each specific domain) (*p* < 0.05). In addition, they were more likely to have severe motor symptoms and lower LNS and BJLO scores, with trend significance (*p* < 0.1) (Table S2).

### 3.3 Risk factors of psychotic symptoms in PD

**FIGURE 2:**
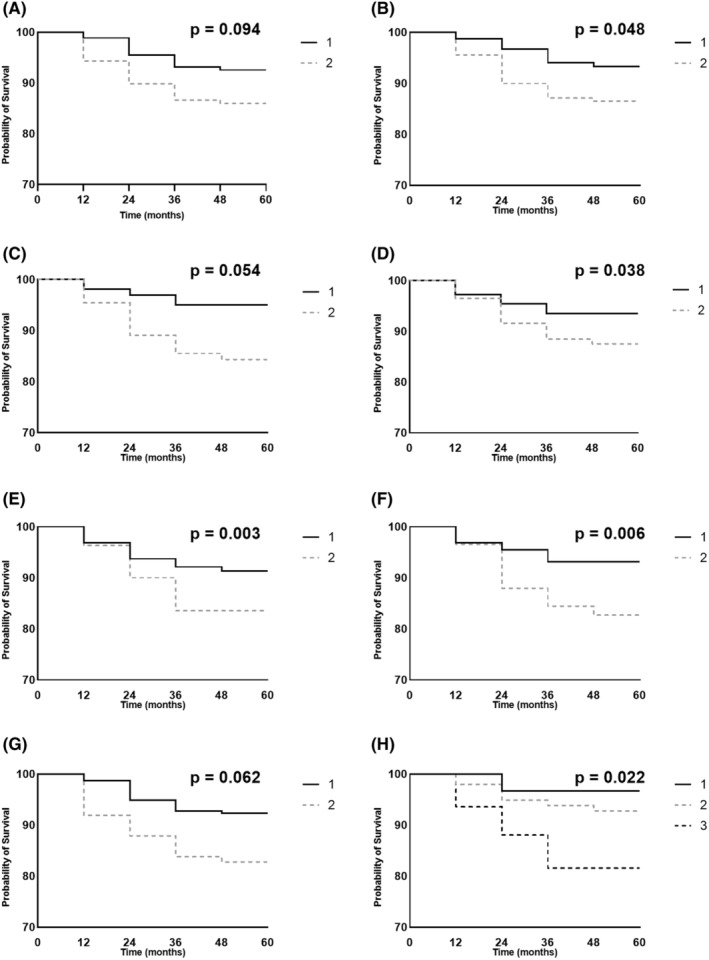
Kaplan‐Meier curves displaying the effects on survival probability of psychotic symptoms in PD for some clinical characteristics. Outcome refers to a score greater than 0 on item 1.2 of the MDS‐UPDRS at two or more assessments. PD patients were dichotomized based on the median sample level of the clinical characteristics. (A) MDS‐UPDRS III (“1” ≤ median and “2” > median), (B) H&Y (“1” represents stage 1 and “2” represents stage 2), (C) HVLT RDI (“1” > median and “2” ≤ median), (D) LNS (“1” > median and “2” ≤ median), (E) pRBD (“1” represents without pRBD and “2” represents with pRBD), (F) SCOPA‐AUT of the gastrointestinal domain (“1” ≤ median and “2” > median), (G) SCOPA‐AUT of pupillomotor (“1” < median and “2” ≥ median), and (H) UPSIT (“1” represents normosmia, “2” represents hyposmia, and “3” represents anosmia). PD, Parkinson's disease; MDS‐UPDRS, Movement Disorders Society‐Unified Parkinson's Disease Rating Scale; MDS‐UPDRS III, Movement Disorders Society‐Unified Parkinson's Disease Rating Scale part III; H&Y, Hoehn and Yahr stage; HVLT RDI, Hopkins Verbal learning Test‐Revised Recognition Discrimination Index; LNS, Letter Number Sequencing; pRBD, probable rapid‐eye‐movement sleep behavior disorder; SCOPA‐AUT, Scales for Outcomes in Parkinson's Disease‐Autonomic symptoms; and UPSIT, University of Pennsylvania Smell Inventory Test.

Kaplan‐Meier curves comparing the cumulative incidence of psychotic symptoms at two or more assessments concerning the MDS‐UPDRS III score, Hoehn and Yahr stage, HVLT RDI, LNS, pRBD, SCOPA‐AUT of the GI domain, SCOPA‐AUT of the pupillomotor domain, and UPSIT score are shown in Figure 2A–H.

As shown in Table 2, multivariate COX regression found greater autonomic symptoms in GI domain (hazard ratio (*HR*) = 1.157, 95% confidence interval (*CI*) 1.022–1.309, *p* = 0.021), and the presence of pRBD (*HR* = 2.596, 95% *CI* 1.287–5.237, *p* = 0.008) at baseline was associated with increased risk of reporting psychotic symptoms at two or more assessments compared to having 0 or 1 psychotic events.

**TABLE 2:** Results of the Cox regression analysis for clinical predictors of psychotic symptoms in PD.

	Univariate analysis			Multivariate analysis		
	*β*	*HR* (95% *CI*)	*p* value	*β*	*HR* (95% *CI*)	*p* value
Age, years	0.013	1.013 (0.977–1.049)	0.483	NA	NA	NA
Sex, %male	−0.609	0.544 (0.255–1.161)	0.116	NA	NA	NA
Disease duration, years	0.029	1.029 (0.978–1.082)	0.268	NA	NA	NA
MDS‐UPDRS III score	0.030	1.031 (0.995–1.068)	0.094	NA	NA	0.272
H&Y (ON)						
H&Y‐1	Reference					
H&Y‐2	0.745	2.107 (1.008–4.407)	0.048*	NA	NA	NA
H&Y‐3	1.864	6.451 (0.825–50.433)	0.076	NA	NA	0.072
Motor subtype						
TD	Reference					
PIGD	0.487	1.627 (0.774–3.418)	0.199	NA	NA	NA
Indeterminate	0.347	1.415 (0.334–6.000)	0.638	NA	NA	NA
Cognitive function						
MoCA score	−0.020	0.980 (0.855–1.123)	0.769	NA	NA	NA
HVLT total recall	−0.016	0.984 (0.954–1.015)	0.300	NA	NA	NA
HVLT RDI	−0.025	0.976 (0.951–1.000)	0.054	NA	NA	0.215
LNS score	−0.124	0.883 (0.785–0.993)	0.038*	NA	NA	0.102
SDMT score	−0.007	0.993 (0.958–1.029)	0.696	NA	NA	NA
SFT score	0.001	1.001 (0.942–1.065)	0.965	NA	NA	NA
BJLO score	−0.045	0.956 (0.903–1.012)	0.118	NA	NA	NA
EDS	0.488	1.629 (0.740–3.586)	0.226	NA	NA	NA
GDS‐15 score	0.095	1.100 (0.896–1.350)	0.362	NA	NA	NA
pRBD, *n* (%)	1.055	2.873 (1.430–5.775)	0.003**	0.954	2.596 (1.287–5.237)	0.008**
SCOPA‐AUT						
Total score	0.013	1.013 (0.982–1.046)	0.409	NA	NA	NA
Gastrointestinal	0.163	1.177 (1.047–1.324)	0.006**	0.146	1.157 (1.022–1.309)	0.021*
Urinary domain	0.023	1.023 (0.966–1.084)	0.441	NA	NA	NA
Cardiovascular	0.217	1.242 (0.818–1.887)	0.310	NA	NA	NA
Thermoregulatory	0.101	1.106 (0.924–1.323)	0.271	NA	NA	NA
Sexual	−0.026	0.974 (0.916–1.036)	0.401	NA	NA	NA
Pupillomotor	0.375	1.455 (0.981–2.156)	0.062	NA	NA	0.367
UPSIT score	−0.047	0.954 (0.916–0.993)	0.022*	NA	NA	0.110

*Note*: Outcome refers to a score greater than 0 on item 1.2 of the MDS‐UPDRS at two or more assessments. Variables with values of *p* < 0.1 in the univariate COX regression analysis and no high correlation (*r* > 0.5) with each other were included in a multivariate model.

Abbreviations: BJLO, Benton Judgment of Line Orientation; ESS, Epworth Sleepiness Scale; GDS, Geriatric Depression Scale; H&Y, Hoehn and Yahr stage; HVLT, Hopkins Verbal learning Test; LNS, Letter Number Sequencing; MDS‐UPDRS, Movement Disorders Society‐Unified Parkinson's Disease Rating Scale; MoCA, Montreal Cognitive Assessment; PD, Parkinson's disease; pRBD, probable rapid‐eye‐movement sleep behavior disorder; RDI, Recognition Discrimination Index; SCOPA‐AUT, Scales for Outcomes in Parkinson's Disease‐Autonomic symptoms; SDMT, Symbol Digit Modalities Test; SFT, Semantic (animal) fluency Test; TD, tremor dominant, PIGD, postural instability/gait difficulty; UPSIT, University of Pennsylvania Smell Inventory Test.

* *p* < 0.05.

** *p* < 0.01.

When we chose to report psychotic symptoms at one or more assessments as an outcome, multivariate COX regression revealed that greater autonomic symptoms in the GI domain (*HR* = 1.207, 95% CI 1.105–1.320, *p* < 0.001) and the presence of EDS (*HR* = 1.897, 95% *CI* 1.118–3.221, *p* = 0.018) at baseline were associated with increased risk, while the presence of RBD had a trend significance (*p* = 0.087) (Table S3).

### 3.4 DAT imaging

Baseline DAT imaging measurements were available for 318 participants, including 31 in the PD‐Psy2+ group. Compared with the PD‐Psy0 group, the PD‐Psy2+ group had a reduced mean putamen SBR (Table 3). In the univariate Cox analysis, the mean putamen SBR was associated with an increased risk of psychotic symptoms at two or more assessments (*HR* = 0.176, 95% *CI* 0.040–0.786, *p* = 0.023).

**TABLE 3:** Baseline DAT imaging and CSF measures in each group.

	PD‐Psy0 (*n* = 255)	PD‐Psy1 (*n* = 48)	PD‐Psy2+ (*n* = 35)	*p* value
DAT imaging (striatal binding ratio)				
Mean caudate	1.96 (0.54)	1.77 (0.51)	1.76 (0.57)	0.069
Caudate asymmetry	1.20 (1.10–1.32)	1.16 (1.10–1.29)	1.19 (1.07–1.28)	0.764
Mean putamen	0.81 (0.66–0.96)	0.79 (0.65–0.92)	0.71 (0.56–0.90)	0.043* (Psy0 < Psy2+)
Putamen asymmetry	1.38 (1.05–1.75)	1.49 (125–1.80)	1.43 (1.12–1.63)	0.574
CSF markers, pg/ml				
Aβ42	368.10 (318.30–412.85)	361.35 (314.65–437.30)	343.40 (297.88–384.60)	0.771
Total tau	40.30 (31.30–49.90)	38.65 (28.95–50.75)	42.00 (33.98–52.78)	0.638
Phosphorylated tau181	12.10 (9.35–17.90)	13.25 (10.10–17.95)	11.45 (9.05–14.50)	0.369
α‐synuclein	1677.26 (1323.75–2203.72)	1841.36 (1391.25–2542.28)	1688.83 (1379.44–1913.11)	0.143
Aβ42: total‐tau ratio	9.20 (7.29–11.66)	9.70 (8.50–11.30)	8.38 (6.18–9.79)	0.208

*Note*: Continuous variables were presented as mean ± standard deviation (SD) or median and interquartile range (IQR). For variables presented as mean (SD), one‐way ANOVA was used. For variable presented as median (IQR), Kruskal‐Wallis test was used.

Abbreviations: CSF, cerebral‐spinal fluid; and Aβ, amyloid β; DAT, dopamine transporter; PD, Parkinson's disease; PD‐Psy0, PD patients with a score of 0 for item 1.2 in the MDS‐UPDRS for each annual assessment during the 5‐year follow‐up period; PD‐Psy1, PD patients with any score >0 at only one assessment; PD‐Psy2+, PD patients with any score >0 at two or more assessments.

* *p* < 0.05.

In a multivariate COX regression model adjusted for the greater autonomic symptoms in the GI domain, the presence of pRBD, and Aβ_42_: t‐tau ratio, the mean putamen SBR was not associated with increased risk of future psychotic symptoms (*p* > 0.05) (Table 4). The similar result was found when we chose to report psychotic symptoms at one or more assessments as an outcome (Table S4).

**TABLE 4:** Results of the Cox regression analyses for biomarker predictors of psychotic symptoms in PD.

	Univariate analysis			Multivariate analysis		
	*β*	*HR* (95% *CI*)	*p* value	*β*	*HR* (95% CI)	*p* value
DAT imaging (striatal binding ratio)						
Mean caudate	−0.542	0.581 (0.304–1.114)	0.102	NA	NA	NA
Caudate asymmetry	−0.413	0.661 (0.089–4.893)	0.686	NA	NA	NA
Mean putamen	−1.735	0.176 (0.040–0.786)	0.023*	NA	NA	0.219
Putamen asymmetry	−0.556	0.574 (0.216–1.522)	0.264	NA	NA	NA
CSF markers, pg/ml						
Aβ42	−0.002	0.998 (0.994–1.002)	0.340	NA	NA	NA
Total tau	0.010	1.010 (0.990–1.032)	0.326	NA	NA	NA
Phosphorylated tau181	−0.008	0.992 (0.947–1.038)	0.725	NA	NA	NA
α‐synuclein	0.000	1.000 (1.000–1.001)	0.578	NA	NA	NA
Aβ42: total‐tau ratio	−0.117	0.890 (0.778–1.018)	0.089	−0.172	0.842 (0.723–0.980)	0.027*

*Note*: Outcome refers to a score greater than 0 on item 1.2 of the MDS‐UPDRS at two or more assessments. Variables with values of *p* < 0.1 in the univariate COX regression analysis and no high correlation (*r* > 0.5) with each other were included in a multivariate COX regression model adjusted for pRBD and score in SCOPA‐AUT gastrointestinal domain.

Abbreviations: Aβ, amyloid β; CSF, cerebral‐spinal fluid; DAT, dopamine transporter; PD, Parkinson's disease.

* *p* < 0.05.

### 3.5 CSF findings

Baseline CSF measurements were available for 244 participants, including 24 from the PD‐Psy2+ group. No differences were found among the three groups in terms of CSF measures (Table 3). In the univariable Cox analysis, the association of Aβ_42_: t‐tau ratio with increased risk of reporting psychotic symptoms at two or more assessments was of borderline significance (*HR* = 0.890, 95% *CI* 0.778–1.018, *p* = 0.089).

In a multivariate COX regression model adjusted for the greater autonomic symptoms in the GI domain, the presence of pRBD, and the mean putamen SBR, Aβ_42_: t‐tau ratio was associated with increased risk of future psychotic symptoms (*HR* = 0.842, 95% *CI* 0.723–0.980, *p* = 0.027) (Table 4). No such association was found when we chose to report psychotic symptoms at one or more assessments as an outcome (Table S4).

### 3.6 Prediction of psychotic symptoms in PD

**FIGURE 3:**
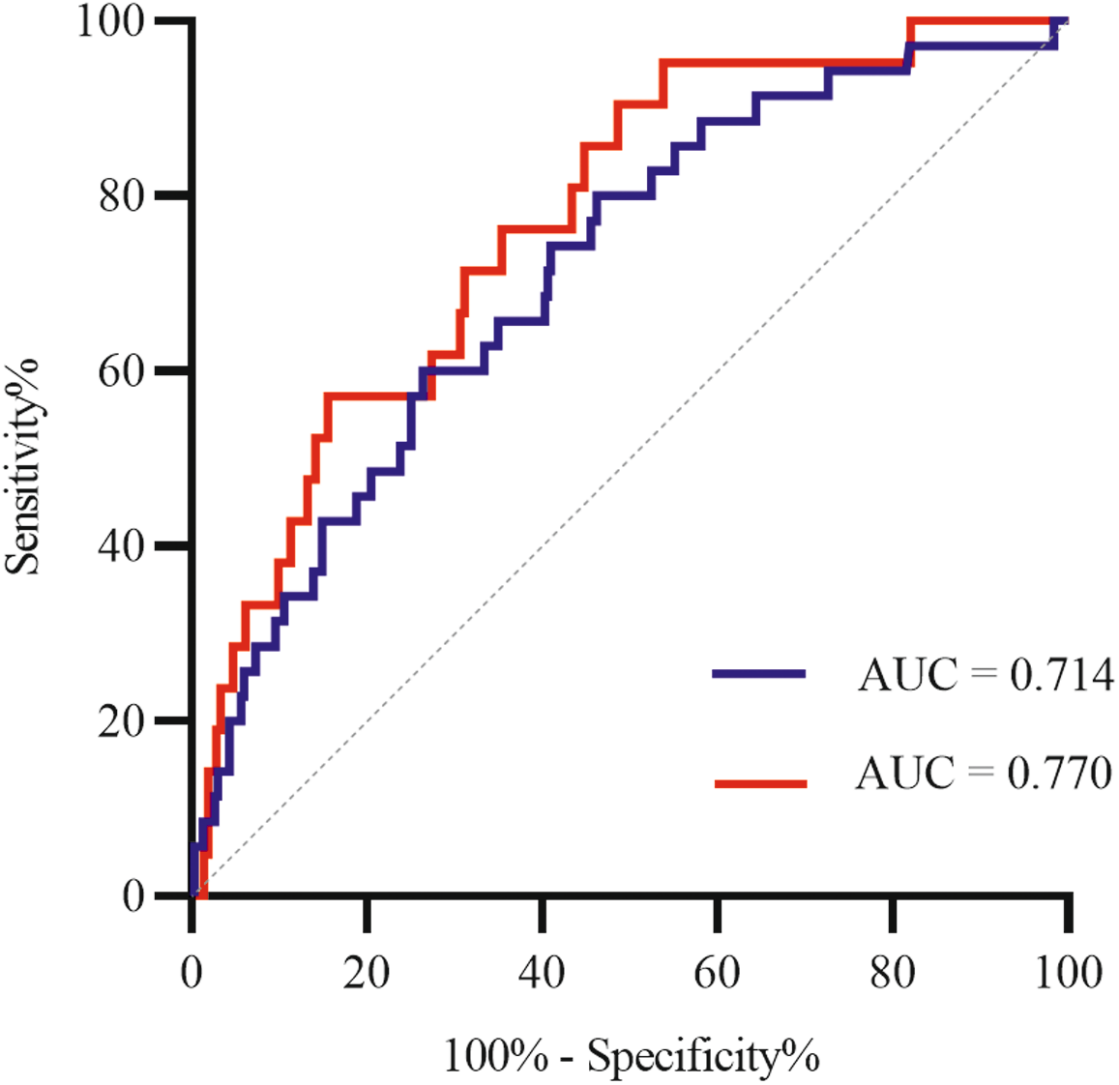
Receiver operating characteristic curve for predicting the emergence of psychotic symptoms during a 5‐year follow‐up in newly diagnosed PD patients (less than 2 years since diagnosis) who did not have psychosis at baseline. Outcome refers to a score greater than 0 on item 1.2 of the MDS‐UPDRS at two or more assessments. The blue line: adjusted for age at baseline, sex, pRBD, and score in SCOPA‐AUT gastrointestinal domain. The red line: adjusted for age at baseline, sex, pRBD, score in SCOPA‐AUT gastrointestinal domain, striatal binding ratio of mean putamen, and Aβ42: total‐tau ratio. MDS‐UPDRS, Movement Disorders Society‐Unified Parkinson's Disease Rating Scale; PD, Parkinson's disease; pRBD, probable rapid‐eye‐movement sleep behavior disorder; SCOPA‐AUT, Scales for Outcomes in Parkinson's Disease‐Autonomic symptoms; and Aβ, amyloid β.

To evaluate the performance of clinical variables (age at baseline, sex, SCOPA‐AUT score in the GI domain, and presence of pRBD), ROC curves were generated (represented with a blue line in Figure 3). The addition of the mean putamen SBR and Aβ42: t‐tau ratio provided a more accurate prediction of future psychotic symptoms than clinical variables alone with a borderline significance (*AUC* = 0.770 [95% *CI* 0.672–0.868] vs. 0.714 [95% *CI* 0.625–0.802], *p* = 0.098) (represented by a red line in Figure 3).

## 4 DISCUSSION

In this study, we identified predictors of psychotic symptoms that developed during the 5‐year follow‐up period in newly diagnosed patients with PD with no psychosis at baseline. Greater autonomic symptoms in the GI domain, presence of pRBD, and Aβ42: t‐tau ratio independently predicted the future development of psychotic symptoms in PD patients. The combined model integrating baseline clinical predictors, DAT imaging, and CSF measures achieved better discrimination ability than clinical predictors alone.

Our study confirmed that autonomic dysfunction and pRBD are risk factors for the future onset of psychotic symptoms in patients newly diagnosed with PD without psychosis at baseline. Previous studies have shown that autonomic dysfunction and pRBD are associated with an increased risk of psychosis in patients with PD.^10^, ^11^, ^16^, ^21^, ^22^, ^23^, ^24^, ^25^, ^26^ However, most of the patients with PD enrolled in those studies had been diagnosed with PD for approximately 5 years. In addition, some previous studies on the PPMI cohort did not exclude patients with PD and psychotic symptoms at baseline.^11^, ^16^ The SCOPA‐AUT GI domain score was associated with early‐onset psychotic symptoms in patients with PD with better sensitivity than the SCOPA‐AUT total score. GI autonomic dysfunction has been proposed as a prodromal non‐motor symptom of PD. Evidence has pointed to abnormal α‐synuclein accumulating not only in the brain but also in the enteric nervous system (ENS).^27^, ^28^, ^29^, ^30^ The accumulation of α‐synuclein in the ENS has been found to correlate with cell death in the autonomic nervous system and is suggested to lead to autonomic dysfunction in the GI domain.^27^ Based on the ENS‐first etiology of PD (the initial α‐synuclein pathology within the ENS),^31^ researchers proposed that the pathologic change first happens in the GI system and then ascends through the vagus nerve to the brainstem which is closely related to the formation of hallucinations.^9^, ^28^, ^32^, ^33^, ^34^ Our findings provide more evidence to support the theory that degeneration of the brainstem is associated with psychosis in PD, and autonomic dysfunction and pRBD in PD may be driven by similar neurobiological mechanisms.

Inconsistent with a previous study of the PPMI cohort, no decrease was found in CSF Aβ42 level in patients with PD with psychotic symptoms,^11^ which may be partly due to sample selection and sample size. Instead, we also found that Aβ42: t‐tau ratio was a risk factor for psychotic symptoms in PD. The ratio of Aβ42: t‐tau has been demonstrated to have predictive ability for mild cognitive impairment for Alzheimer's disease (AD) in previous studies,^35^, ^36^ which means Aβ42: t‐tau ratio is a biomarker that can indicate AD pathology earlier than Aβ42 level. PD psychosis in later disease stages has been linked to higher levels of amyloid, tau, and α‐synuclein pathology in frontal, parietal, and hippocampal areas.^37^ However, the exact link between AD pathology and early‐onset PD psychosis remains poorly understood. The decreased Aβ42: t‐tau ratio might explain the poor cognitive function outcomes at the final assessment in PD patients with psychotic symptoms.

Our study had several strengths. First, we evaluated the predictors of early‐onset PD psychosis in patients with PD without psychotic symptoms at baseline in a relatively large sample. Data were collected from a longitudinal cohort study. Second, we investigated clinical, DAT imaging, and CSF predictors separately and in combination. Clinical characteristics included systematic data on motor and non‐motor symptoms, genetic information, and peripheral inflammatory biomarkers. Baseline clinical markers had discriminative ability with an *AUC* of 0.714, suggesting that the development of psychotic symptoms 5 years after PD diagnosis can be predicted with good accuracy with clinical markers alone. The addition of the mean putamen SBR and Aβ42: t‐tau ratio provided a more accurate prediction of future psychotic symptoms than clinical variables alone with a trend significance, with an *AUC* of 0.77. Third, we used the SCOPA‐AUT total score and scores from the six different autonomic function domains for the analysis. We found that the SCOPA‐AUT GI domain score may be more specific than the total SCOPA‐AUT score.

Our study has some limitations. First, the evaluation of psychotic symptoms in the PPMI was limited to a single item in the MDS‐UPDRS, assessing the occurrence of psychotic symptoms within 1 week, which did not meet the PD psychosis criteria.^38^ One research suggested that the MDS‐UPDRS item 1.2 score ≥1 highly correlated with the SAPS score with 42% sensitivity and 96% specificity for identifying psychosis in PD.^25^ Besides, to improve specificity, we defined any score >0 at two or more assessments as an outcome in this study. Second, we did not include PD medication in the analysis. Evaluating the impact of medications on psychotic symptoms is complex due to the variety of drugs involved and their interrelated effects.^39^ The interaction between medication use and psychotic symptoms is dynamic and changes over time for individuals, with the relationship being bidirectional. Considering these challenges and our study's focus on baseline predictors, along with our statistical methodology, we chose not to account for medication effects in our research. Third, pRBD was assessed using subjective self‐report questionnaires, which could be prone to measurement errors. In the future, objective measures like polysomnography could be utilized. However, due to time constraints and technological limitations, these methods are often not feasible for large‐scale population studies.

In conclusion, the present study found that patients with PD with more GI autonomic dysfunction, pRBD, and a decreased Aβ42: t‐tau ratio were at a higher risk of developing early‐onset psychotic symptoms. Our study provides additional evidence and implications for prognostic stratification and therapeutic approaches in PD psychotic spectrum disorders.

## AUTHOR CONTRIBUTIONS

Jing Chen and Baoyu Chen were the co‐first authors of this work. Concept and design: Jing Chen, Xiaotong Feng, Lin Zhang, and Junliang Yuan. Collection and assembly of data: Jing Chen, Xiaotong Feng, Danhua Zhao, Yuan Li, Junyi Chen, Chaobo Bai, Xintong Guo, and Xiaoyu He. Data analysis and interpretation: Jing Chen, Baoyu Chen, Xiaotong Feng, Danhua Zhao, Qi Wang, and Lin Zhang. Manuscript writing: All authors. Final approval of manuscript: All authors. The final version has been revised by: Junliang Yuan, Jing Chen, Baoyu Chen, Xiaotong Feng, and Lin Zhang.

## FUNDING INFORMATION

This study was supported by the National Natural Science Foundation of China (82071552, 22376006) and the Chinese Academy of Sciences Grant (JCTD‐2021‐06).

## CONFLICT OF INTEREST STATEMENT

The authors declare that they have no competing interests.

## Supporting information


Tables S1–S4.

## Data Availability

The data that support the findings of this study are available from the first author or the corresponding author upon reasonable request.
