# Supplementary material for: Predictors for early‐onset psychotic symptoms in patients newly diagnosed with Parkinson's disease without psychosis at baseline: A 5‐year cohort study
Source: CNS Neurosci Ther. 2024 Mar 3;30(3):e14651. doi: 10.1111/cns.14651 (PMC10909617; doi:10.1111/cns.14651)
Supplement: Supplementary file 1 — Tables S1–S4. [file CNS-30-e14651-s001.docx]

**Predictors for psychotic symptoms in newly diagnosed Parkinson’s disease patients free from psychosis at baseline: a 5-year cohort study**

**Supplementary tables**

**Table S1** Results of univariate COX regression analysis of GBA status, APOE ε4 status, and Peripheral inflammation biomarkers

**Table S2** Clinical characteristics of patients with PD at the fifth-year follow-up

**Table S3** Results of the Cox regression analysis for clinical predictors of psychotic symptoms in PD

**Table S4** Results of the Cox regression analysis for biomarker predictors of psychotic symptoms in PD

**Table S1 Results of univariate COX regression analysis of GBA statue, APOE ε4 status, and Peripheral inflammation biomarkers**

|  | Univariate analysis^a^ | | | Univariate analysis^b^ | | | |  |
| --- | --- | --- | --- | --- | --- | --- | --- | --- |
|  | *β* | *HR* (95%*CI*) | *p* value |  | *β* | *HR* (95%*CI*) | *p* value | |
| *GBA* status |  |  |  |  |  |  |  | |
| *GBA* negative |  | Reference |  |  |  |  |  | |
| *GBA* mutation | 0.121 | 1.128 (0.624–2.039) | 0.689 |  | 0.019 | 1.019 (0.395–2.627) | 0.968 | |
| *APOE* ε4 status | | | | | | | |  |
| *APOE* ε4 negative |  | Reference |  |  |  |  |  | |
| *APOE* ε4 heterozygous | 0.076 | 1.079 (0.646–1.803) | 0.770 |  | 0.206 | 1.228 (0.576–2.622) | 0.595 | |
| *APOE* ε4 homozygous | -0.078 | 0.925 (0.128–6.667) | 0.938 |  |  | NA |  | |
| Peripheral inflammation | | | | | | | |  |
| neutrophil | 0.053 | 1.054 (0.917–1.213) | 0.458 |  | -0.035 | 0.965 (0.756–1.233) | 0.777 | |
| lymphocyte | 0.521 | 0.867 (0.562–1.339) | 0.521 |  | -0.020 | 0.980 (0.536–1.827) | 0.949 | |
| lymphocyte-to-neutrophil ratio | -1.002 | 0.367 (0.104–1.291) | 0.118 |  | -0.441 | 0.643 (0.107–3.871) | 0.630 | |

^a^ Outcome refers to a score greater than 0 on item 1.2 of the MDS-UPDRS at one or more assessments. ^b^ Outcome refers to a score greater than 0 on item 1.2 of the MDS-UPDRS at two or more assessments.

Abbreviations: PD, Parkinson’s disease; GBA, β-glucocerebrosidase; APOE, apolipoprotein (*APO*) *E* status; MDS-UPDRS, Movement Disorders Society–Unified Parkinson Disease Rating Scale.

**Table S2 Clinical characteristics of patients with PD at the fifth-year follow-up**

|  | PD-Psy0  (n = 255) | PD-Psy1  (n = 48) | PD-Psy2+  (n = 35) | *p* value |
| --- | --- | --- | --- | --- |
| Age, years | 66.08 (9.90) | 66.96 (8.82) | 67.57 (8.12) | 0.618 |
| Sex, %male | 157 (61.6%) | 25 (52.1%) | 26 (74.3%) | 0.121 |
| Disease duration, years | 8.00 (6.75–11.00) | 7.00 (6.00–14.00) | 8.00 (6.00–14.00) | 0.840 |
| MDS-UPDRS III score | 23.88 (13.80) | 24.50 (14.51) | 30.15 (15.40) | 0.052 |
| H&Y (ON) | |  |  |  |
| H&Y-1 | 44 (17.8%) | 5 (10.9%) | 4 (12.5%) |  |
| H&Y-2 | 182 (73.7%) | 37 (80.4%) | 21 (65.6%) |  |
| H&Y-3 | 13 (5.3%) | 3 (6.5%) | 4 (12.5%) | 0.138 |
| Motor subtype | |  |  |  |
| TD | 94 (36.9%) | 13 (27.1%) | 117 (34.7%) |  |
| PIGD | 135 (52.9%) | 23 (67.6%) | 23 (67.6%) |  |
| indeterminate | 26 (10.2%) | 4 (8.3%) | 1 (2.9%) | 0.324 |
| Cognitive function | | | | |
| MoCA score | 28.00 (26.00–29.00) | 27.00 (24.00–28.00) | 27.00 (25.25–28.75) | 0.009^**^  (Psy0 > Psy2+) |
| HVLT total recall | 44.19 (18.79) | 41.24 (21.26) | 38.32 (20.14) | 0.215 |
| HVLT RDI | 55.00 (44.75–56.00) | 56.00 (44.00–56.00) | 50.00 (44.75–55.75) | 0.023^*^  (Psy0 > Psy2+) |
| LNS score | 10.00 (9.00–13.00) | 10.00 (8.00–11.00) | 10.00 (8.00–11.00) | 0.098 |
| SDMT score | 40.89 (17.73) | 38.11 (19.83) | 35.09 (18.59) | 0.188 |
| SFT score | 12.00 (8.00–16.00) | 11.00 (7.00–15.00) | 10.50 (7.50–16.00) | 0.842 |
| BJLO | 26.00 (24.00–28.00) | 24.00 (22.00–26.00) | 25.00 (21.00–29.50) | 0.053 |
| ESS score | 7.00 (4.00–11.00) | 7.00 (4.00–11.00) | 7.00 (5.25–11.50) | 0.006^**^  (Psy0 < Psy2+) |
| GDS-15 score | 5.00 (5.00–6.00) | 5.00 (4.00–6.00) | 5.50 (4.00–7.00) | 0.105 |
| pRBD, n (%) | 126 (49.6%) | 35 (72.9%) | 27 (84.4%) | <0.001^**^  (Psy0 < Psy1, Psy0 < Psy2+) |
| SCOPA-AUT score | | | | |
| Total score | 14.00 (9.00–23.00) | 14.00 (9.00–6.00) | 20.00 (13.25–29.75) | <0.001^**^  (Psy0 < Psy1, Psy0 < Psy2+) |
| Gastrointestinal | 3.00 (2.00–5.00) | 3.00 (2.00–4.00) | 4.50 (3.25–6.75) | <0.001^**^  (Psy1 < Psy2+, Psy0 < Psy2+) |
| Urinary domain | 5.00 (3.00–7.00) | 5.00 (3.00–6.00) | 6.00 (5.00–7.75) | 0.002  (Psy1 < Psy2+, Psy0 < Psy2+) |
| Cardiovascular | 0.00 (0.00–1.00) | 0.00 (0.00–1.00) | 0.00 (0.00–1.75) | 0.038^*^  (Psy0 < Psy2+) |
| Thermoregulatory | 1.00 (0.00–2.00) | 1.00 (0.00–4.00) | 1.50 (0.25–3.00) | 0.001^**^  (Psy0 < Psy1, Psy0 < Psy2+) |
| Sexual | 2.00 (1.00–6.00) | 3.00 (2.00–12.00) | 4.00 (1.00–14.75) | 0.078 |
| Pupillomotor | 0.00 (0.00–1.00) | 1.00 (0.00–1.00) | 0.50 (0.00–1.75) | 0.008^**^  (Psy0 < Psy1, Psy0 < Psy2+) |
| UPSIT score | 17.50 (13.00–23.00) | 15.00 (11.00–26.00) | 15.00 (11.00–26.00) | 0.600 |

^*^*p* <0.05, ^**^*p* <0.01.

Continuous variables were presented as mean ± standard deviation (SD) or median and interquartile range (IQR). Categorical variables were presented as numbers and percentages. For variables presented as mean (SD), one-way ANOVA was used. For variables presented as number (percent), chi-square test was used. For variable presented as median (IQR), Kruskal-Wallis test was used.

Abbreviations: PD, Parkinson’s disease; PD-Psy0, PD patients with a score of 0 for item 1.2 in the MDS-UPDRS for each annual assessment during the 5-year follow-up period; PD-Psy1, PD patients with any score >0 at only one assessment; PD-Psy2+, PD patients with any score >0 at two or more assessments; MDS-UPDRS, Movement Disorders Society–Unified Parkinson Disease Rating Scale; H&Y, Hoehn and Yahr stage; TD, tremor dominant; PIGD, postural instability/gait difficulty; MoCA, Montreal Cognitive Assessment; HVLT, Hopkins Verbal learning Test; RDI, Recognition Discrimination Index; LNS, Letter Number Sequencing; SDMT, Symbol Digit Modalities Test; SFT, Semantic (animal) fluency Test; BJLO, Benton Judgment of Line Orientation; ESS, Epworth Sleepiness Scale; GDS, Geriatric Depression Scale; pRBD, probable rapid-eye-movement sleep behavior disorder; SCOPA-AUT, Scales for Outcomes in Parkinson’s Disease-Autonomic symptoms; UPSIT, University of Pennsylvania Smell Inventory Test.

**Table S3 Results of the Cox regression analyses for clinical predictors of psychotic symptoms in PD**

|  | Univariate analysis | | |  | Multivariate analysis | | |
| --- | --- | --- | --- | --- | --- | --- | --- |
|  | *β* | *HR* (95% *CI*) | *p* value |  | *β* | *HR* (95% *CI*) | *p* value |
| Age, years | 0.009 | 1.009 (0.987–1.033) | 0.415 |  | NA | NA | NA |
| Sex, %male | 0.014 | 1.014 (0.652–1.577) | 0.952 |  | NA | NA | NA |
| Disease duration, years | 0.037 | 1.037 (1.004–1.072) | 0.028^*^ |  | NA | NA | 0.111 |
| MDS-UPDRS III score | 0.030 | 1.031 (1.007–1.055) | 0.011^*^ |  | NA | NA | 0.212 |
| H&Y (ON) | |  |  |  |  |  |  |
| H&Y-1 | Reference | | |  |  |  |  |
| H&Y-2 | 0.399 | 1.490 (0.948–2.343) | 0.084 |  | NA | NA | NA |
| H&Y-3 | 0.779 | 2.178 (0.297–15.978) | 0.779 |  | NA | NA | 0.387 |
| Motor subtype | |  |  |  |  |  |  |
| TD | Reference | | |  |  |  |  |
| PIGD | 0.362 | 1.437 (0.870–2.372) | 0.157 |  | NA | NA | NA |
| indeterminate | 0.630 | 1.878 (0.809–4.361) | 0.142 |  | NA | NA | NA |
| Cognitive function | | | | | | | |
| MoCA score | 0.015 | 1.015 (0.925–1.113) | 0.755 |  | NA | NA | NA |
| HVLT total recall | -0.010 | 0.990 (0.970–1.010) | 0.329 |  | NA | NA | NA |
| HVLT RDI | -0.013 | 0.987 (0.970–1.005) | 0.153 |  | NA | NA | NA |
| LNS score | -0.071 | 0.931 (0.861–1.007) | 0.076 |  | NA | NA | 0.676 |
| SDMT score | -0.006 | 0.994 (0.971–1.017) | 0.591 |  | NA | NA | NA |
| SFT score | 0.009 | 1.009 (0.970–1.050) | 0.657 |  | NA | NA | NA |
| BJLO | -0.022 | 0.978 (0.940–1.019) | 0.294 |  | NA | NA | NA |
| EDS | 0.758 | 2.135 (1.310–3.477) | 0.002^**^ |  | 0.640 | 1.897 (1.118–3.221) | 0.018^*^ |
| GDS-15 score | 0.093 | 1.098 (0.961–1.253) | 0.169 |  | NA | NA | NA |
| pRBD , n (%) | 0.658 | 1.931 (1.234–3.023) | 0.004^**^ |  | NA | NA | 0.087 |
| SCOPA-AUT | | | | | | | |
| total score | 0.020 | 1.021 (1.001–1.041) | 0.042 |  | NA | NA | NA |
| Gastrointestinal | 0.162 | 1.176 (1.089–1.269) | <0.001^**^ |  | 0.189 | 1.207 (1.105–1.320) | <0.001^**^ |
| Urinary domain | 0.021 | 1.022 (0.984–1.061) | 0.265 |  | NA | NA | NA |
| Cardiovascular | 0.287 | 1.332 (1.027–1.727) | 0.030^*^ |  | NA | NA | 0.425 |
| Thermoregulatory | 0.087 | 1.091 (0.968–1.229) | 0.154 |  | NA | NA | NA |
| Sexual | 0.002 | 1.002 (0.969–1.037) | 0.893 |  | NA | NA | NA |
| Pupillomotor | 0.178 | 1.195 (0.894–1.596) | 0.228 |  | NA | NA | NA |
| UPSIT score | -0.032 | 0.969 (0.944–0.995) | 0.018^*^ |  | NA | NA | 0.132 |

^*^*p* <0.05, ^**^*p* <0.01.

Outcome refers to a score greater than 0 on item 1.2 of the MDS-UPDRS at one or more assessments. Variables with values of *p* <0.1 in the univariate logistic regression analysis and no high correlation (*r* >0.5) with each other were included in a multivariate COX regression model.

Abbreviations: PD, Parkinson’s disease; MDS-UPDRS, Movement Disorders Society-Unified Parkinson Disease Rating Scale; H&Y, Hoehn and Yahr stage; TD, tremor dominant, PIGD, postural instability/gait difficulty; MoCA, Montreal Cognitive Assessment; HVLT, Hopkins Verbal learning Test; RDI, Recognition Discrimination Index; LNS, Letter Number Sequencing; SDMT, Symbol Digit Modalities Test; SFT, Semantic (animal) fluency Test; BJLO, Benton Judgment of Line Orientation; ESS, Epworth Sleepiness Scale; GDS, Geriatric Depression Scale; pRBD, probable rapid-eye-movement sleep behavior disorder; SCOPA-AUT, Scales for Outcomes in Parkinson’s Disease-Autonomic symptoms; UPSIT, University of Pennsylvania Smell Inventory Test.

**Table S4 Results of the Cox regression analyses for biomarker predictors of psychotic symptoms in PD**

|  | Univariate analysis | | |  | Multivariate analysis | | |
| --- | --- | --- | --- | --- | --- | --- | --- |
|  | *β* | *HR* (95% *CI*) | *p* value |  | *β* | *HR* (95% *CI*) | *p* value |
| DAT imaging (striatal binding ratio) | | | | | | | |
| mean caudate | -0.454 | 0.635 (0.419–0.964) | 0.033^*^ |  | NA | NA | 0.286 |
| caudate asymmetry | -0.739 | 0.477 (0.125–1.824) | 0.280 |  | NA | NA | NA |
| mean putamen | -0.914 | 0.401 (0.167–0.964) | 0.041^*^ |  | NA | NA | NA |
| putamen asymmetry | -0.106 | 0.900 (0.528–1.532) | 0.697 |  | NA | NA | NA |
| CSF markers | | | | | | | |
| Aβ_42_ | -0.001 | 0.999 (0.996–1.002) | 0.546 |  | NA | NA | NA |
| Total tau | 0.009 | 1.009 (0.995–1.023) | 0.190 |  | NA | NA | NA |
| phosphorylated tau_181_ | 0.001 | 1.001 (0.975–1.029) | 0.924 |  | NA | NA | NA |
| α-synuclein | 0.000 | 1.000 (1.000–1.001) | 0.004^**^ |  | 0.000 | 1.000 (1.000–1.001) | 0.007^**^ |
| Aβ_42_: total-tau ratio | -0.075 | 0.928 (0.853–1.010) | 0.084 |  | NA | NA | 0.590 |

^*^*p* <0.05, ^**^*p* <0.01.

Outcome refers to a score greater than 0 on item 1.2 of the MDS-UPDRS at one or more assessments. Variables with values of *p* <0.1 in the univariate logistic regression analysis and no high correlation (*r* >0.5) with each other were included in a multivariate COX regression model adjusted for EDS and score in SCOPA-AUT gastrointestinal domain.

Abbreviations: PD, Parkinson’s disease; DAT, dopamine transporter; CSF, cerebral-spinal fluid; Aβ, amyloid β; MDS-UPDRS, Movement Disorders Society-Unified Parkinson Disease Rating Scale; EDS, excessive daytime sleepiness; SCOPA-AUT, Scales for Outcomes in Parkinson’s Disease-Autonomic symptoms.
